# A promising method for identifying cross-cultural differences in patient perspective: the use of Internet-based focus groups for content validation of new Patient Reported Outcome assessments

**DOI:** 10.1186/1477-7525-4-64

**Published:** 2006-09-22

**Authors:** Mark J Atkinson, Jan Lohs, Ilka Kuhagen, Julie Kaufman, Shamsu Bhaidani

**Affiliations:** 1 Worldwide Health Outcomes Research, La Jolla Laboratories, Pfizer Inc., San Diego, CA 92121, USA; 2 Health Services Research Center, USCD School of Medicine, La Jolla, CA 92093, USA; 3 Lohs Research Group, Qualitative Marketing Research, 2170 West Freeman Road, Palatine, IL 60067, USA; 4 IKM International Qualitative Marketing Research, Ludwig-Ganghoferstr. 33, D-85551 Kirchheim/München, Germany; 5 Kaufman Associates, 6 Fennwood Drive, Atherton, CA 94027, USA; 6 President and Chief Technical Officer, FocusForums™, Calgary, Alberta T3K 6J1, Canada

## Abstract

**Objectives:**

This proof of concept (POC) study was designed to evaluate the use of an Internet-based bulletin board technology to aid parallel cross-cultural development of thematic content for a new set of patient-reported outcome measures (PROs).

**Methods:**

The POC study, conducted in Germany and the United States, utilized Internet Focus Groups (IFGs) to assure the validity of new PRO items across the two cultures – all items were designed to assess the impact of excess facial oil on individuals' lives. The on-line IFG activities were modeled after traditional face-to-face focus groups and organized by a common 'Topic' Guide designed with input from thought leaders in dermatology and health outcomes research. The two sets of IFGs were professionally moderated in the native language of each country. IFG moderators coded the thematic content of transcripts, and a frequency analysis of code endorsement was used to identify areas of content similarity and difference between the two countries. Based on this information, draft PRO items were designed and a majority (80%) of the original participants returned to rate the relative importance of the newly designed questions.

**Findings:**

The use of parallel cross-cultural content analysis of IFG transcripts permitted identification of the major content themes in each country as well as exploration of the possible reasons for any observed differences between the countries. Results from coded frequency counts and transcript reviews informed the design and wording of the test questions for the future PRO instrument(s). Subsequent ratings of item importance also deepened our understanding of potential areas of cross-cultural difference, differences that would be explored over the course of future validation studies involving these PROs.

**Conclusion:**

The use of IFGs for cross-cultural content development received positive reviews from participants and was found to be both cost and time effective. The novel thematic coding methodology provided an empirical platform on which to develop culturally sensitive questionnaire content using the natural language of participants. Overall, the IFG responses and thematic analyses provided a thorough evaluation of similarities and differences in cross-cultural themes, which in turn acted as a sound base for the development of new PRO questionnaires.

## Article overview

We begin this article with two brief literature reviews: One to identify how Internet focus groups (IFG) have been used in health and social science research; the second to examine current approaches to cross-cultural validation of PROs. Based on these growing bodies of knowledge, there appeared compelling reasons to extend IFG based methods to assist with the cross-cultural adaptation of new patient-reported outcome measures. As a result, a proof of concept (POC) study was specifically designed to assess the usefulness of IFG-based inquiry to detect and explore thematic differences across linguistically and culturally different peoples. This POC study was conducted in Germany and the United States, and involved persons experiencing problems with oily skin of the face and scalp.

More specifically, the qualitative IFG methods involved the thematic coding of multi-lingual transcripts, which in turn provided comparative thematic data between countries; these results were used to adapt the content of candidate items for a series of new PRO measures. Moderators' implementation of coding and thematic analysis activities involved a significant change in their traditional roles; which also required their more formal involvement as members of the PRO design team. Greater use of moderators in PRO content development activities is a good use of expertise, due to their deep emersion in, and understanding of, the concerns and cultural perspectives expressed by participants.

## Review 1: Internet focus groups a new technology

The use of Internet technologies as a medium for social 'dialogue' has become tremendously popular over the last decade. The transformation of text-based bulletin-board services into multimedia 'blogs' and virtual community networks have lead to a proliferation of both formal and informal discussion groups which address almost any topic imaginable. A specialized form of virtual interest group is used for consumer research, the Internet Focus Group (IFG); also known as bulletin board focus groups in the US [1]. IFGs first appeared in the late 1990's and have since been used by educators, clinicians, researchers and marketing specialists to research stakeholder values [2], explore cross-cultural differences [3], and provide supportive and educational on-line environments [4,5]. Within healthcare delivery research, IFGs have also been used to better understand patients' perspectives and knowledge of their disease conditions and/or medical treatments (1). All of which has given rise to various research organization specializing in the use of virtual methodologies (see for example: [6-9]).

Despite some sampling concerns associated with the use of IFG technology among less affluent or older persons, the use of IFGs as a marketing and research tool continues to grow. This is likely due to a number of practical reasons, three of the most important are: 1) The ability to overcome geographical and physical restrictions to participation; 2) the ease and speed of participant engagement, facilitation and surveying; and 3) the automated management of resulting transcripts and survey data [4]. Demonstration that virtual methods provide equivalent qualitative results as both traditional face-to-face and telephone methodologies has also furthered the use of IFGs in mainstream research [10,11]. Moreover the quality of results from IFGs may be greater than face-to-face methods when addressing topics of a sensitive nature, and participants often report feeling freer to provide candid responses (with less social desirability bias) than would be the case in face-to-face settings [12-15]. Table 1 presents a more detailed summary of potential advantages and some limitations of IFG use.

**Table 1:** Benefits and Limitations of Internet based Focus Groups

	**Potential Benefits**	**Potential Limitations**
**Recruitment and Scheduling**	- Wide geographical recruitment allows access to socially or geographically isolated participants and the inclusion of persons with uncommon concerns - Internet-based recruitment sources (clinical databases, advocacy associations, and on-line support groups) permit rapid enrollment - Recruitment is made easier by flexible participation requirements (times, locations and travel) - Typing speed is not essential, as participants type at their own pace	- Limited computer experience or access can restrict participation, leading to age or socio-economic sampling bias - The identity of participants cannot be easily verified - Technical requirements of the IFG application for specific browser software may limit participation and should be assessed at screening - Respondents with certain medical conditions or inpatient treatment settings may not be able to participate
		
**Facilitator Role as IFG Moderator**	- Email eases the communication between focus group facilitators and participants (directives, reminders, and follow-ups) - Software management functions monitor the IFG sessions (on-line tracking of activities and participation levels) - Polling functions allow facilitators to sample opinions and use these results within IFG sessions - Reference libraries store surveys and multimedia files or historical documentation for use as later reference materials and within the IFG sessions themselves	- Facilitators may spend more time on-line than for an equivalent series of face-to-face focus groups
		
**Participant Responses**	- Perceptions of anonymity allow for greater comfort when discussing sensitive issues - Responses are less redundant since respondents can read and consider the ideas of others before replying. - Participants can take their time when responding to questions, leading to considered responses and high-quality data - 'Emoticons' and customizable text message formats can be used to express feeling or act in place of non-verbal cues	- Redundant information may be generated if the lines of questioning in the Topic Guide are too general or vague - Reduced opportunity to refine or clarify responses may result in the use of leading or restrictive lines of inquiry
		
**Facilitator Role as Co-investigator**	- Facilitators' professional role can be expanded to include thematic research activities, including content analysis of IFG responses - Session transcripts are immediately available and permit drill-down comparison of phraseology and evaluation of topical content - Poll and survey results can be used to examine qualitative and thematic differences by participant characteristics and opinions - Multi-cultural implementations of IFGs allow bilingual facilitators to participate in parallel cross-cultural development activities based on their great familiarity with the concerns and issues expressed by participants within the sessions	- More time and care is required to formulate questions and probes to be used in the Topic Guide - Moderator training may be required on such qualitative topics as; 'Grounded Theory' and thematic content analyses - Preparation and modification of thematic coding schedules require a clear (but modifiable) conceptual framework and consistent coding practice. For some applications, evaluation of the degree of agreement between coders may be required (inter-rater reliability)
		
**Time & Costs of Project Execution**	- Costs associated with collection and content analysis of IFG responses are less than one-on-one interviewing - On-line transcripts and use of automated thematic coding functions organize thematic analyses and generation of thematic frequency counts - No additional costs are associated with conducting IFGs that cover wide geographical areas - There are no moderator and client travel expenses	- Reimbursement costs to IFG participants may be higher than traditional focus groups due to the increased time spent on-line - Greater facilitator costs are likely due to a major role expansion and increased facilitator involvement [70], which are off-set by reduced transcription and project management costs

## Review 2: Cross-cultural validation of patient reported outcomes

Borrowing psychometric methods developed in psychology, Outcomes Research (OR) scientists develop reliable and valid measures to assess the impact of clinical conditions and medical interventions from the patients' perspective. Early in the design phase of new Patient Reported Outcome (PRO) measures, patients are involved in content validation activities to identify meaningful themes and dimensions of future measurement. Typically, patient focus groups or interviews help assure that: 1) The content of new measures adequately cover concerns and issues which are important to patients/consumers; 2) The wording of new questions and instructions are based in the natural language and phraseology of respondents; and 3) The instructions, item pool, and response options are understandable and acceptable to persons who will be completing the surveys.

Over the years, the essential process of content validation has been included as a central topic in various PRO guidance documents authored by PRO outcomes working groups and drug regulatory agencies [16-27]. More recently, an additional set of recommendations regarding PRO content was made by membership of the 1999 Health Outcomes Methodology Symposium; "...that measurement tools be... more culturally appropriate for diverse populations and more conceptually and psychometrically equivalent across such groups"[28]. In response to such calls for culturally sensitivity and relevance, instrument developers have begun to address cultural content issues when designing new patient-reported measures: Some examples include; epidemiological surveys [29], clinical assessment and screening tools [30,31], and community health surveys [32].

Various methods have been tried to reduce the cultural content bias of PROs. By far the most common is to follow rigorous procedures to adapt an instrument designed in one culture for use in other cultural contexts. Guidelines for such cross-cultural adaptation activities are well defined (see IQOLA and ERIQA guidelines [33,34]) and rely on a rigorous forward and backward translation methodology [35,36], followed by the use of psychometric replication (or bridging) studies to examine the internal and external validity of the 'adapted' translation in the target culture [37]. A much less frequently used approach involves the use of thematic review and harmonization of content between focus groups conducted concurrently in different cultures, a method known as parallel cross-cultural PRO content validation [38]. This approach has been tried by relatively few instrument developers [39-41], largely due to the time and budgetary resources associated with the initial stages of questionnaire design.

Unfortunately, it is rare during cultural adaptation of PRO measures to include the re-validation of the content coverage in the target culture. While biological and clinically assessed indicators are often considered more universal in nature, the manifestation and impact of disease and disability on the lives of individuals is typically culturally bound. Nevertheless, an implicit assumption is often made that the original thematic content and scale dimensions are equally relevant across all cultures. As a result, various academics have argued that culturally unique content may be missed during the adaptation processes, and that input from patients in different target cultures is necessary to design instruments with adequate coverage of unique cultural meaning [36,42]. The failure to assess the cultural limitations of existing item content can result in culturally adapted measures with poor 'ecological validity' (i.e., the measure is ill suited to the context) and which do not address culturally-specific concerns [43-45].

When cultural differences in content or content relevance are identified after the fact, there are several approaches to handle such discrepancies. Some instrument developers have chosen to use only those items which are relevant across all cultural contexts and thus the re-validated measure is intended to possess a universal scale structure. An example of such an approach was taken during recent revisions to the Women's Health Questionnaire (WHQ) where developers made a decision to remove items that exhibited signs of cultural specificity [46]. Another approach is to use more general wording for items, which removes references to culturally specific content and allows individuals greater latitude when interpreting what situations the questions refer to [47,48]. The EQ-5D is a well-known example of a PRO that uses general summary items to assure perceived relevance across cultures and across illness conditions [49]. Another, rarely used, solution is to allow the specific item content to vary in each different culture [31]. This approach requires significant content redevelopment activities for each country in which the PRO is applied. Table 2 presents an overview of the various ways instrument designers help ensure the cross-cultural validity of PRO content.

**Table 2:** Cross-cultural content development solutions used during PRO development

**Options for Cross-Cultural Harmonization of PRO Content**	**Benefits**	**Indicators of a Problem**
**Option 1**: Address cultural issues using a rigorous translation and testing process for item content developed in a single source country	Initial PRO content design is less time-consuming since attempts to revalidate in different cultures does not involve patient reassessment of PRO content	- Poor face validity and complaints that the PRO does not address cultural issues (cultural bias) - Differences in measure performance across cultures are difficult to explain and require use of statistical patches to address such differences - Entanglement of disease, treatment and cultural effects
**Option 2**: Use content-specific items that are identified as equally relevant across all cultures	May work well for assessment of physical manifestations of disease and treatment since these are often similar across cultures	- Content may seem to duplicate clinical information gleaned through patient-reports - The impacts of illness and treatment on the psychological and social domains of life may not be fully characterized
**Option 3**: Use more generally worded (domain) summary items that allow for interpretation based on respondents' cultural perspective	- Good estimation of the general impact of illness and treatment across cultures - Comparable domain estimates across cultural settings	- Uncertainty about what cultural and disease-specific events respondents are referring to when making summary ratings
**Option 4**: Use a different set of content-specific items for each culture	Measures are high relevance in the cultural settings where item content was developed	- Duplication of content validation and psychometric development is required for each country - Assessment results may not be comparable across countries if item difficulty is not equivalent
**Option 5**: Use a blend of all item types, which may include: 1. A set of culturally-specific items 2. A set of content-specific items relevant across all cultures 3. A set of general summary items	- High cultural relevance of the resulting measure - The general impacts of disease and treatment effects are comparable across cultures - Ability to evaluate the relative importance of specific item content with the cultural context using rating on general summary items	- Requires careful planning and execution of cross-cultural content validation studies - The tasks associated with item and scale design may be more complex than for other options although, following construct validation, the resulting measures may not be more complex or burdensome

Internet Focus Group technologies may provide a way to address long-standing concerns about PRO content development based on geographically and culturally limited sampling. A major advantage of IFGs over traditional face-to-face focus groups is they extend the researcher's ability to span geographical barriers within the constraints of limited project resources. Moreover, they may provide a way to use a set of standardized procedures and tools for cross-cultural harmonization of content during early PRO development. As yet, however, the usefulness of IFGs for cross-cultural use has not been systematically evaluated.

## Proof of concept study: IFGs and cross-cultural PRO content development

**Figure 1:**
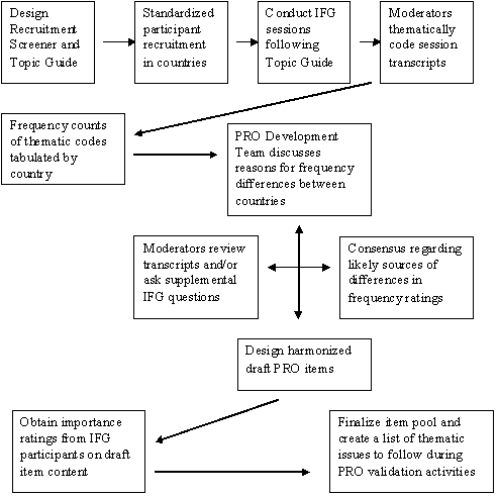
A flow diagram of the stages of IFG cross-cultural content validation process.

This POC study was part of a larger project to develop and validate a new set of PROs that assess the symptomatic impact of oily skin on the face (and scalp) among patients in the US and Germany. The concepts we sought to demonstrate were that IFGs methods can be used to identify differences in thematic content between countries and that such inquiry can lead to a better understanding of the various reasons for such differences. It was anticipated that prior knowledge of thematic differences could be fruitfully applied during the cross-cultural development of new PROs. Figure 1 presents a diagrammatic overview of the major activities occurring over the course of the POC study.

### Recruitment of participants

US and German IFG participants were recruited using standard methods, namely, from patient/consumer databases of individuals willing to take part in market research. These databases are maintained by market research companies specifically for such purposes. Some additional participants were recruited by asking database referrals to suggest others they know with similar problems (oily skin). In the US, a small number of participants (n = 4) were recruited from prior face-to-face focus groups addressing patients' concerns and experiences with oily skin.

Potential recruits between the ages of 18 and 65 years were screened by telephone using a Recruiting Questionnaire (i.e., the Screener) and those who met the following criteria were invited to participate:

1. All participants were required to:

     • Perceive portions of their face or their scalp to be oily

     • Experience that their oily skin/scalp was bothersome

     • Actively and regularly attempt to control the level of facial/scalp oiliness

2. A proportion of the samples also included individuals who experienced the following:

     • Mild or moderate acne

     • Seen a dermatologist in the past 2 years for their acne

     • An oily scalp and were also balding (males only)

     • Represented Asian, Black, Latino/Hispanic, White/Caucasian peoples

     • Represented various regions of the country (US only)

### IFG methods and thematic analysis

The current consumer-based POC study used an on-line IFG application called FocusForums™ to explore how individuals with oily skin characterize and evaluate both the symptoms and impact of their condition on their daily lives. This IFG application contains a number of functions to assist with development and refinement of content for the new PRO item pool (see Table 3).

**Table 3:** IFG functions and their use during PRO content development

**FUNCTION**	**Description of Function**	**Use During PRO Content Development**
**Text Based Session Transcripts**	All transcripts, including moderator questions/probes, participant responses, and external observer comments, are available on-line and can be made searchable by thematic content.	Participants' responses were revisited to: - Explore reasons for content differences between the countries - Assure that PRO item wording and phraseology used natural language
**Qualitative Coding Function**	The qualitative coding function allows moderators to create hierarchical coding categories with an unlimited number of sub-categories. Participant responses can then be thematically coded for later retrieval and summarization. The ability to add coding comments to the coded items for later reference.	Responses were coded into one or more coding categories, from which frequency counts identified common themes which could be further sub-grouped by focus group members' characteristics (such as country, gender, or disease characteristics).
**Fish-Bowl Function**	The fish bowl or backroom function allows observers to make comments regarding participants' posts. These (color coded) comments are visible only to moderators and other external observers.	Moderators used this function to integrate the comments/queries from external IFG observers (e.g., members of the PRO development team, KOLs) during sessions to guide probing of participant responses during the sessions.
**Document Management Areas**	File management areas are used to store the most recent versions of materials such as participant screeners, topic guide, and the qualitative coding schedule.	On-line documentation provided planners and research analysts with current versions of all relevant study documents for the purposes of updating, discussion, and later reference.
**Multilingual Implementation**	The integrated language modules of FocusForums™ allow moderators to conduct all IFG activities in the users' native language.	Moderators had access to transcripts and thematic frequency results in their native language. These resources were used to identify cross-cultural similarities and differences, as well as make content and wording recommendations during the design of the new questionnaires.

A Topic Guide was developed to flexibly guide the lines of inquiry within the IFGs. This guide was based on a conceptual model arising from a literature review and input from dermatology thought leaders. Over the course of four days, focus group members participated on-line for approximately 45 minutes each day – during which they provided written responses to questions contained in the Topic Guide, follow-up probes from moderators, and the comments of other participants. The thematic content of these responses (i.e., the transcripts) were independently coded by the US and German moderators using a draft Thematic Coding Schedule. When a response did not seem fit in any of the existing coding categories, the moderator created a new coding category to categorize and tag the new thematic content. The primary purpose of this modifiable Coding Schedule was to identify content differences between the sets of IFGs conducted in the two countries. Once content differences were identified, reasons for these differences could be explored; some of which could be attributable to the effects of culture.

Table 4 presents a truncated example of the frequency counts of the number of unique individuals who made comments in each of the thematic coding categories. Great skill and patience was required of the moderators to read and code the large number responses (over 770 US and 1040 German responses), each response often contained a number of subtly inter-related themes, in such cases multiple codes were applied. The involvement of moderators in this coding task was a significant alteration in their usual qualitative activities.

**Table 4:** Frequency counts of unique respondents making comments in various coding categories related to the daily management of skin oiliness*

**Coding Class**	**Total Sample** **(n = 54)**	**US Sample** **(n = 28)**	**German Sample** **(n = 26)**
**Appearance and Social Impact**			
Perception of appearance	67% (36/54)	71% (20/28)	62% (16/26)
Self-consciousness**	59% (32/54)	46% (13/28)	73% (19/26)
Social Confidence	18% (10/54)	18% (5/28)	19% (5/26)
Distress/Interruption			
Preoccupation appearance**	56% (30/54)	28% (8/28)	85% (22/26)
Worry about need to manage condition**	31% (17/54)	21% (6/28)	42% (11/26)
Frequency checking skin oiliness	18% (10/54)	14% (4/28)	23% (6/26)
Impact on Daily life			
Washing or Cleansing for oil control**	65% (35/54)	75% (21/28)	54% (14/26)
Times of day when typically washing	44% (24/54)	46% (13/28)	42% (11/26)
Need to Blot**	41% (22/54)	64% (18/28)	15% (4/26)
Apply Face Powder (females only) **	52% (14/27)	38% (5/13)	64% (9/14)
Makeup (Re)Application (females only)**	30% (8/27)	54% (7/13)	7% (1/14)
*Number of cleansings per day*			
- 1–2	42% (23/54)	36% (10/28)	50% (13/26)
- 3–5	50% (27/54)	39% (11/28)	62% (16/26)
- 6–15	18% (10/54)	11% (3/28)	27% (7/26)
*Effect on diet*			
- No Fast Food, No Rich Food	54% (29/54)	50% (14/28)	58% (15/26)
- No Chocolate, No Sweets**	26% (14/54)	7% (2/28)	46% (12/26)
- Eat Healthy Foods, Eat More Fruit**	26% (14/54)	36% (10/28)	15% (4/26)

* Coding categories which were used to code 15% or less of the overall participants were dropped.

** Categories where differences between German and US frequencies were observed.

As indicated by '**' coding categories in Table 4, some thematic codes were applied more frequently in one of the two countries. These differences were discussed during teleconferences between the IFG moderators and the PRO Development team. Moderators, drawing on their first-hand experience within the IFG sessions, lead the discussion about how such differences in thematic endorsement might be explained. Table 5 presents the possible reasons for observed differences in the coding frequencies between the two countries and the questions that need to be addressed in order to evaluate each of these reasons.

**Table 5:** Potential reasons for observed differences in the numbers of people endorsing a particular theme

**Potential Reason for Thematic Differences**	**Questions to consider and discuss**
The IFG participants differed between countries in terms of recruitment sources and/or sample characteristics	• Are there any systematic differences in sample characteristics between the two countries? • Are the sampling differences a result of cultural differences in the larger population or are they due to differences in recruitment approaches? • Do the different coding frequencies make sense based on known sample composition?
	
IFG moderators followed different lines of qualitative inquiry to gather information	• Were there differences in the numbers and types of probes used by moderators for the particular topic? • Were there differences in the number and types of supplemental questions asked from other sources (e.g., session observers)?
	
The Coding Schedule was applied in different ways by the moderators	• Did the moderators apply different coding categories to a particular type of response? If so, what was the reasoning behind their approach to coding? • Did existing or newly created categories overlap with other coding categories?
	
The observed differences might be due to cultural differences	• Did the observed frequency differences between countries result from differences in the ways respondents understood or described their condition? • Did the ways respondents behaved or coped with their condition differ significantly? • When discussing all the possible reasons for the observed differences, did cultural or social factors seem plausible?

### Sample selection

Differences in sample characteristics of the focus groups could have lead to differences in how the participants elaborated and explored topical issues. In turn, such differences could have affected how responses were ultimately coded. Although a standardized recruitment screener was used to help assure that the composition of IFG membership was consistent across countries, some sampling differences may have been culturally unavoidable. For example in this study, the samples of US and German IFGs differed on their medical treatment histories. IFG participants in Germany reported more medical consultations for their condition than those in the US. This may have been due to differences in access/use of health service delivery systems in the two countries or differences in the severity of the condition itself.

### Session dynamics

During cross-cultural harmonization discussions, it was determined that some differences in coding frequency arose from variation in the number and types of probing questions used by the IFG moderators. While the moderators used the same Topic Guide to facilitate the IFGs, they used additional probes to develop a more comprehensive understanding of certain issues and behaviors. The practice of spontaneous probing is wholly consistent with qualitative research methodologies [50]. These probing questions were not prearranged, but rather emanated from the unique dynamics and flow of discussion within the particular IFG. In response to supplemental questioning, IFG members likely made additional comments and because these probes were not applied equivalently across groups and countries, the frequencies of certain thematic categories were unequally represented. An example of differential probe use can be seen in the Distress/Interruption sub-section of Table 5, where US and German coding frequencies differed on "preoccupation with appearance". Such differences should not be automatically assumed to represent a true cultural difference.

### Transcript coding

Other differences in content frequencies may have been due to how moderators decided to code participants' responses. Decisions about how to classify a particular response were not always clear-cut and were based on coder interpretation. In such instances, moderators made independent judgments about which coding categories to assign to responses. Since coding categories were occasionally changed in response to what was observed within the response transcripts, reliance on inter-rater reliability analyses and coder retraining (an often used exploratory research method) was not considered a useful focus in this study. Moreover, the primary purpose of the content coding activity was to highlight areas for discussion, not to focus on the reliability of the coding schedule itself [51]. An example occurred when a modification of the German coding schedule was made to account for a distinction between oiliness of the 'side of nose' versus the 'nose', the US moderator on the other hand, used only the 'nose' code to characterize both types of responses. When such distinctions were encountered during harmonization discussions, moderators evaluated the potential reasons for distinctions and typically agreed to collapse categories where differences were not thought to be culturally determined.

### Cross-cultural differences

A final explanation for the differences in thematic frequency counts relates to the distinctive linguistic, conceptual, and experiential differences which exist between the two cultures. For example, differences in the use of *dry blotting *versus *wet blotting *codes lead to a further review of the original transcripts in this area. It was determined that dry blotting was preferred by US females because, unlike wet blotting, this method of facial oil control did not require them to reapply their make-up foundation. On the other hand, German females, who mentioned fewer make-up concerns and a greater reliance on facial powder to control the appearance of oily skin (shine), seemed less concerned by washing; possibly due to the relatively straightforward task of reapplying facial powder. Possibly providing some support for this notion, both US and German males (who did not report using make-up) indicated that they washed the face with soap and water more often than female participants.

Another potential area of cultural difference was the mention of eating behaviors as a way of reducing skin oiliness. The moderators suggested that the German culture may foster a mindset of "avoidance" of things that might be harmful; while those in the US may tend to believe they can prompt favorable outcomes by being proactive and engaging in positive behavior. This working hypothesis arose out of the observation that German participants more frequently indicated they attempted to control excess sebum by avoiding "bad" things such as chocolate and sweets; whereas US participants more frequently indicated that their skin would be less oily if they did "good" things such as eating "healthy foods." Such differences may reflect cultural differences in how individuals understood and approached the daily management of their condition.

### PRO item design

Following harmonization discussions to identify potential areas of cultural differences, PRO item pools were developed based on the most commonly occurring coding themes. During item design, the original IFG transcripts were revisited to assure that wording, phraseology and concepts in the new assessments reflected those used by the focus group participants in each country. Once the questions for the new oily skin scales were drafted, the IFG participants were invited back to provide cognitive debriefing feedback and to rate the degree to which the proposed items addressed important aspects of their condition. The item importance ratings provided yet another opportunity to assess cultural differences in the relative importance of item content and how items might perform differently between the two countries in the future. Table 6 provides an example of importance rating results for a new set of "Symptom Bother" rating scales.

**Table 6:** Importance rating of symptom bother items by country (ordered from most to least important)

*Item*	*All Grps Mean*^++^	*US*	*Mean^++^* *(SD)*	*Deutsch*	*Mean^++^* *(SD)*	*F value*	*P Value*
Unattractive	1.6	1.7	(1.0)	1.4	(0.7)	1.37	0.25
Frustrated	2.0	1.9	(1.1)	2.1	(1.1)	0.50	0.49
Inconvenienced	2.1	2.1	(1.1)	2.1	(0.9)	0.01	0.94
Bothered	2.1	2.1	(1.0)	2.2	(1.0)	0.15	0.70
Embarrassed	2.1	1.8	(1.0)	2.0	(1.1)	5.43	0.02*
Nervous	2.2	2.5	(1.3)	2.0	(0.8)	2.24	0.14
Discouraged	2.2	1.9	(1.0)	2.7	(1.1)	5.83	0.02*
Annoyed	2.2	2.0	(1.1)	2.5	(0.9)	3.06	0.09
Disgusted	2.2	2.0	(1.1)	2.4	(1.3)	0.98	0.33
Self-conscious	2.2	1.6	(1.0)	2.8	(1.4)	10.75	0.00***
Preoccupied/Distracted	2.3	2.3	(1.0)	2.4	(0.9)	0.04	0.85
Worried	2.4	2.3	(1.2)	2.5	(1.2)	0.49	0.49
Irritable	2.4	2.4	(1.3)	2.4	(1.2)	0.03	0.86
Distressed	2.5	2.4	(1.1)	2.7	(1.1)	0.64	0.43

^++ ^1, Extremely important; 2, Very Important; 3, Important; 4, A Little Important; 5, Not important at all.

* p < .05

*** p < .001

The largest difference in importance ratings of these rating scales occurred on the 'self-conscious' item, with German IFG participants indicating the term was much less important than the US participants. This 'relevancy' or 'importance' rating difference suggests that the cross-cultural performance of this item in particular should be subject to closer inspection during later construct validation activities. Interestingly, self-consciousness was also singled-out by a professional PRO translation services as a term that was difficult to translate into German.

## Discussion

The use of IFGs for parallel cross-cultural PRO content development was both time/cost effective and received very positive reviews from participants. The thematic frequency analysis of IFG transcripts highlighted a number of areas of difference between countries, which led to fruitful discussion within the content harmonization sessions. Various explanations were explored which could account for observed differences, including both non-cultural factors (e.g., the effects of, sampling, probing, coding) as well as cultural factors. Occasionally, the discussions prompted a re-review of the original transcripts as new cultural and gender issues were raised and considered. Information about the most commonly endorsed thematic categories and potential areas of thematic difference between cultures provided a solid basis on which to draft PRO questions; a draft that reflected the common concerns and issues of IFG participants. The proposed questions, were then reviewed by participants and rated as to their importance. The resulting importance ratings provided further clues as to which items might differentially perform across cultures in future studies.

### IFGs and the changing roles of the professional moderator

In the past, the role of professional moderators has addressed the largely independent mandate to conduct qualitative inquiry within focus groups sessions. Once moderators identified the major focus group themes and issues which seem important, these themes and issues were then summarized in a final focus group report. Typically, the involvement of moderators ended as they passed this report on to the PRO development teams responsible for preparing the draft PRO item pools and construct validation activities. In the current study, moderators were much more active in instrument design activities, particularly the thematic coding and frequency analyses. It is informative to review some of the philosophic and methodological tensions that moderators may encounter as they take on this new role. Tensions which also seem to exist between various schools of thought about research methodologies in the health sciences, social science, and field of applied marketing [52-55].

When qualitative focus groups are used to validate the content of new PRO measures, either explicitly or implicitly, the investigative methods used by two different epistemologies come into contact. These ways of gleaning 'truth' can be characterized as belonging to either a qualitative tradition, based on an inductive and phenomenological approach; or a quantitative tradition, based on a deductive and positivistic approach [53,54]. By nature, qualitative focus group research is *inductive*, open-ended and flexible, responding to the flow of each unique session, rather than closed-ended and fixed. Consistent with various qualitative research methods, the focus group inquiry allows the patients the freedom to provide information that does not necessarily fit with any expectation/hypotheses going into the research. It is precisely this openness to new and unexpected information that allows measurement designers to more fully "ground" the content of new Patient Reported Outcomes in the concerns and issues that patients think are relevant [56].

In turn, PRO design specialists use this deeper understanding of patient themes and issues to design pools of questions that measure the relevant content [57] and the performance of new assessment scales are evaluated in subsequent psychometric studies. These later psychometric studies utilize quantitative (statistical) methods to reduce the length and detail of surveys so that they only measure the most important concepts to most respondents. The resulting measurement scales allow for the qualitative assessment of predetermined concepts – an approach which appears to run counter to principles of qualitative inquiry. Supporting the distinction between qualitative and quantitative methods, Brookes suggests that qualitative methods are used to validate conceptual meaning using phenomenological data (an inductive approach) and quantitative validation activities focus on measurement and operational activities associated with the hypothetical deductive approaches of positivistic science [58].

### When qualitative and quantitative activities meet

The apparent duality between qualitative and quantitative methods, however, may not be clear cut and some have argued that both inductive and hypothetical-deductive methods of inquiry may compliment each other [59-64], or at least provide similar results [65]. Supporting a blending of traditions, advocates of most qualitative schools of thought acknowledge that any inquiry is influenced to some degree by the interests and understanding of the interviewer, as well as the objectives of their qualitative work. In order to account for such influences, qualitative research methods often include self-reflective activities where the interviewer identifies their own influences on the processes of qualitative exploration and interpretation [66].

Parallels can be drawn between the influence of moderator's personal knowledge on the direction of qualitative inquiry and the influence of a body of knowledge in a particular field on what is explored within a focus group ([67], pp. 92–4). Indeed, current PRO development guidelines recommend that instrument design start by defining a clear 'conceptual framework', developed with input from key clinical opinion leaders' who have experience understanding patient perspectives and a good understanding of applied outcomes research [68,69]. The 'conceptual framework' should not be confused with a 'conceptual' or 'theoretical model', whose organization is based on a set of predefined and empirically testable relationships. The conceptual framework is a way of sketching out the current understanding in a particular area of interest and forms the basis for development of the Discussion/Topic Guide used to guide IFG inquiry. The conceptual framework is then modified through qualitative inquiry according to what does and does not make sense to patients, as well as what aspects of patients' experiences, perspectives and behaviors have not been taken into account by the initial framework.

Early in the current study, moderators expressed concerns that the Topic Guide and coding activities lead to a quantitative reduction and over-simplification of qualitative findings. Questions arose as such as: 'Do we lead the lines of inquiry too much?' 'Does the detailed coding activities focus too much on the detail versus the bigger picture?' and 'Is it really necessary for the moderators to perform the coding functions?'. Such questions reflect initial concerns as exemplified by the follow statement made by one of the moderators:

"I personally have struggled considerably with the coding. I found it an enormous "stretch" as a qualitative researcher, who tries to see the "big picture in the constellation in the stars," versus focusing on small details."

Over time, however, moderators began to see practical value in thematic analysis as they explored the reasons for differences in thematic endorsement between the two countries. Recursive discussion about the various thematic differences resulted in more expansive ways of describing observations and distinguishing cultural differences from other sources of thematic variation – resulting in a deeper understanding of cultural issues and perspectives. The re-review of previously coded transcript materials provided substantive examples of how in-depth analysis of responses was stimulated by analysis of coding frequency results.

### What could have been done differently?

Moderators had a number of suggestions about what could have been done differently in the future and their suggestions provide some direction for refinement of the methodology.

#### Keep coding activities simple

The complexity of the themes covered in this proof of concept study presented a particular challenge. The coding schedule was too long and required subdividing across the four different IFG sessions. This gave rise to concerns that responses to open-ended questions asked on one day contained information that should have been coded in a different part of the schedule. If the same schedule were used across all sessions, the topical coverage might have to be reduced.

#### Implement the coding schedule in a timely fashion

It was recommended that the coding activities be preformed at the end of each day and that results of the frequency analyses allow moderators to ask follow-up questions during the following IFG session. This would have provided a more informative way to directly probe participants' views on thematic differences. In order to speed the coding activities, it was suggested that an independent bilingual coder be employed to reduce the interpretive demands placed on the moderators.

#### Alternatives to a coding approach

In order to reduce problems associated with differential probing patterns between moderators, a 'cross-reader' approach was suggested as an alternative to the thematic coding and frequency analysis. This reader could simply read responses, looking for differences, or alternately read and code responses in a more consistent manner. One of the moderators stated:

"Based on our experiences, if you have moderators who speak at least 2 of the languages, you can cross read. Then you can do the real qualitative work with the guide only, and let it flow, probe and dig deeper. These issues could then be communicated and synchronized <harmonized>."

A less popular alternative to cross-reading was the suggestion that more structure be imposed on the use of probes within the IFG sessions. Since moderators often used in-session probes to address questions raised by session observers, spontaneous use of probes in one group would have to be implemented across other sessions, a proposition which was thought unwieldy.

## Concluding remarks

In summary, the qualitative activities of IFGs appear to be enhanced through the use of thematic analyses which help to focus moderator discussion on topics associated with cross-cultural differences in thematic content. In this proof of concept study, the methods were shown to work, although some refinement of approaches may help simplify the tasks without compromising the usefulness of IFGs for cross-cultural harmonization.

Coding is an additional tool that can help moderators summarize and quickly compare the level of thematic endorsement between countries and between IFGs within a country. If applied in a timely manner (same day and subsequently) the thematic coding results can facilitate further exploration within the next IFG session. Such results also support the process of cross-cultural harmonization of issues, as facilitators re-visit responses and compare similar statements of different respondents in light of new information about potential group and cultural differences. The method however, is not intended as a substitute for qualitative inquiry itself, and the process of understanding the thoughts, experiences and values of the customer. IFGs and thematic analysis are additional tools in the professional toolbox of focus group moderators.
